# Physio-Microstructural Properties of Aerated Cement Slurry for Lightweight Structures

**DOI:** 10.3390/ma11040597

**Published:** 2018-04-12

**Authors:** Areej T. Almalkawi, Talal Salem, Sameer Hamadna, A. G. N. D. Darsanasiri, Parviz Soroushian, Anagi Balchandra, Ghassan Al-Chaar

**Affiliations:** 1 Department of Civil and Environmental Engineering, Michigan State University, 3546 Engineering Building, E. Lansing, MI 48824, USA; 2 Metna Corporation, 1926 Turner Street, Lansing, MI 48906-4051, USA; 3 Construction Engineering Research Laboratory (CERL), US Army Engineer Research and Development Center, Champaign, IL 61822, USA

**Keywords:** aerated cement slurry, foaming agent, microstructure, thermal conductivity, sorptivity, density, compressive strength, lightweight structures, composites

## Abstract

Cementitious composites, including ferrocement and continuous fiber reinforced cement, are increasingly considered for building construction and repair. One alternative in processing of these composites is to infiltrate the reinforcement (continuous fibers or chicken mesh) with a flowable cementitious slurry. The relatively high density of cementitious binders, when compared with polymeric binders, are a setback in efforts to introduce cementitious composites as lower-cost, fire-resistant, and durable alternatives to polymer composites. Aeration of the slurry is an effective means of reducing the density of cementitious composites. This approach, however, compromises the mechanical properties of cementitious binders. An experimental program was undertaken in order to assess the potential for production of aerated slurry with a desired balance of density, mechanical performance, and barrier qualities. The potential for nondestructive monitoring of strength development in aerated cementitious slurry was also investigated. This research produced aerated slurries with densities as low as 0.9 g/cm^3^ with viable mechanical and barrier qualities for production of composites. The microstructure of these composites was also investigated.

## 1. Introduction

Continuous fiber reinforced cement composites and ferrocement are examples of composites used in building construction and repair applications [1,2,3,4]. When compared with polymer composites, cementitious composites offer improved fire and moisture resistance, and economics. The density of cementitious matrices, however, is higher than that of polymeric matrices. 

One approach to production of cementitious composites involves infiltration of the reinforcement system with a cementitious slurry. Aeration of the slurry offers the opportunity to reduce the density of cementitious matrices. While aeration tends to compromise the strength of cementitious materials, the aerated matrices may still be able to meet the demands on their mechanical performance in the context of composites with relatively high-volume fractions of continuous reinforcement with high specific surface area. These demands are different from those placed on concrete in conventional reinforced concrete structures. The combination of aeration and reinforcement of high specific surface area and close spacing could also provide desired workability attributes (e.g., ease of screw application and cutting) that would make some wood construction techniques applicable to the material.

Efforts to reduce the density of slurry need to address (besides mechanical performance and interactions with different reinforcement systems such as wire mesh reinforcement) two aspects of the slurry behavior that are of practical significance in this application: (i) sorptivity for protecting the insulation and the interior of the building against moisture transport; and (ii) thermal conductivity for adding value towards enhancing the energy-efficiency of the building. 

The synergy between fibers and organic polymers has been key to the emergence of composites as widely used structural materials. In this synergistic action, fibers account for the distinctly high strength and modulus of composites. Organic polymers, on the other hand, provide for stress transfer to fibers, and stress redistribution among fibers upon early rupture of some statistically weaker ones [5]. These contributions of the polymer matrix rely heavily upon their strain capacity and desired adhesion to fibers. Given the brittle nature of polymer matrices such as epoxy; it is their relatively low elastic modulus that is responsible for their elongation capacity. The polymer matrix also contributes barrier qualities to composites. The generally open molecular structures of both organic polymers and fibers are responsible for the relatively low density of composites that benefits their ‘specific’ strength and modulus. Weight saving has been a vital consideration in transition from metals to composites in aerospace and other applications [5].

The work reported herein focuses on development of an inorganic matrix with reduced density and elastic modulus that suits utilization as matrix in continuous fiber reinforced composites that have similarities to ferrocement products. The matrix developed here is an aerated slurry that offers desired rheological attributes for infiltrating fabric and mesh reinforcement systems. Aerated cementitious materials (e.g., aerated concrete) have been developed mostly to provide thermal insulation qualities [6,7,8]. This work focused on the development of aerated slurries with a relatively low density and modulus and viable strength for use as matrix in structural composites as investigated in our previous studies [9,10,11,12,13]. This aerated slurry-infiltrated mesh is developed as a construction material that offers qualities intermediate between wood and concrete. It is intended to provide a desired balance of relatively low density, ductility, toughness, strength, workability, moisture and fire resistance, and durability under weathering exposure.

## 2. Materials and Methods

**Figure 1:**
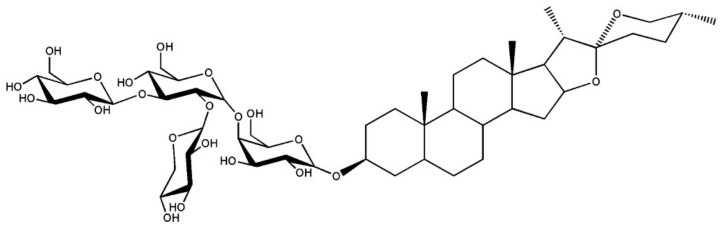
Saponin molecule extracted from sisal waste [16].

The aerated slurry was prepared by mixing the foaming agent (saponin). Saponins are natural surfactants found abundantly in various plant species. More specifically, they are amphipathic glycosides comprising one or more hydrophilic glycoside moieties combined with a lipophilic triterpene derivative [14]. Figure 1 shows a saponin molecule obtained from the residues of sisal defibering. It has been used in formulation of detergents [15].

**Figure 2:**
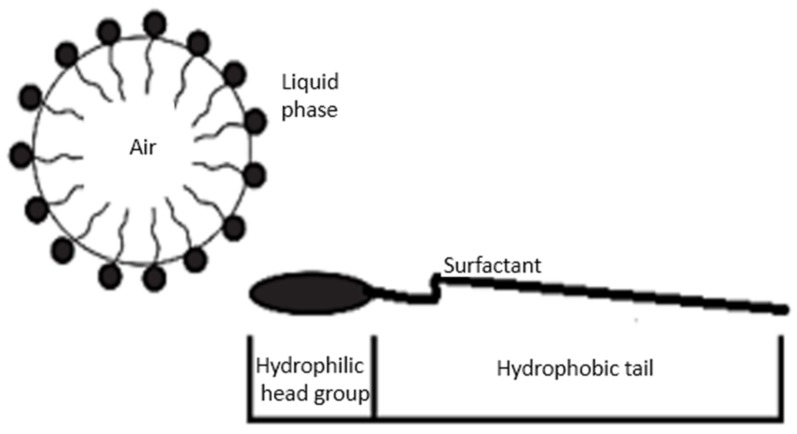
Surfactant molecules at the water–air interface [25,30].

**Figure 3:**
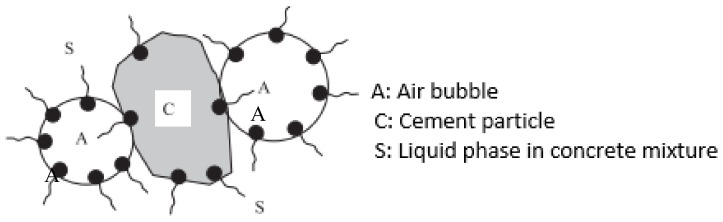
Interaction between air bubbles and cement particles [25].

Aeration of cementitious materials can be accomplished via stabilization of entrapped air using surfactants [17,18,19], or by the addition of fine powder that generates gas by undergoing chemical reactions with cementitious materials. Aerated concrete has been produced with a wide range of densities (300 to 1800 kg/m^3^). The focus of this work is on achieving densities below 1000 kg/m^3^ that are generally viewed as insulating materials [20,21]. Saponin (a hydrolyzed protein extracted from plants) [22,23,24] was used in this work as a surfactant for production of aerated slurry. Saponin was mixed with the mixing water of slurry, and agitated to form foam, which was then mixed with Portland cement type 1 to produce the aerated slurry. As a surfactant, saponin lowers the surface tension of water. Surfactants are molecules with polar and nonpolar ends that attach to water and air, respectively. Surfactants are molecules with polar and nonpolar ends that attach to water and air, respectively. The orientation of surfactant molecules in bulk solution is random. Those occurring at the air/liquid interfaces or adsorbed on cement particles, however, have preferred orientations that tend to minimize the unfavorable interactions between the liquid phase and different molecular sections of the surfactant. Figure 2 shows the alignment of a monolayer of surfactant molecules at the interface between air and surrounding liquid phase. The hydrophobic tails of surfactant molecules stick out of the solution to reduce the distortion of water molecules, and thus lower the overall free energy of the system [25,26]. The mutual repulsion between the hydrophilic heads of surfactant molecules reduces the attraction of the bulk liquid phase, producing a lower surface tension. Because of the electrostatic component of the repulsion force of ionic surfactants, their effectiveness to reduce surface tension is more significant than that provided by nonionic surfactants [27]. The nature and concentration of surfactants determine the physical and chemical properties of the air bubble/liquid interfaces, including surface tension (equals to free surface energy) and stability. The electrostatic and steric repulsions between surfactants helps to stabilize the air bubbles that form within the liquid [28,29]. The hydrophilic ends of the surfactant molecules are also electrostatically attracted to cement particles, this is also a factor in stabilizing the air bubbles within cement paste (Figure 3).

**Figure 4:**
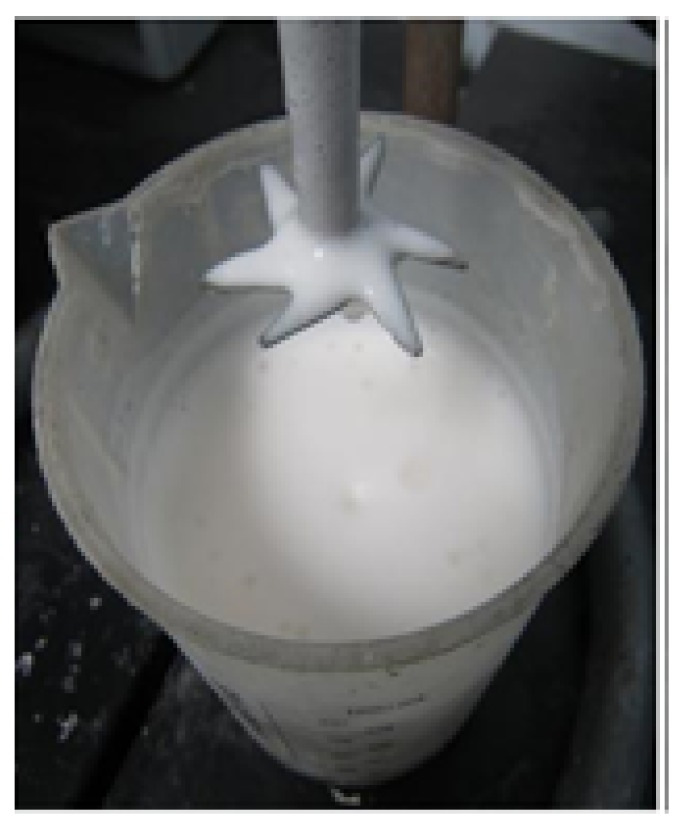
Foam generation in water via high-speed mixing.

Saponin and water were mixed at 1200 rpm rotational speed, using a Craftsman^®^ mixing blade attached to a drill (Figure 4), to produce foamed water. The foamed mixing water was then added to cement at water/cement ratio of 0.45 to 0.6. Mixing was accomplished in a mortar mixer for 2 min. The water/cement ratio of different aerated slurries was adjusted in order to produce a desired fresh mix rheology for infiltrating multiple layers of chicken mesh. The required fresh mix rheology could be defined by a viscosity of about 1900 cP and a yield strength of about 70. The resulting aerated slurry was placed in 50 mm cube molds and kept in sealed condition at room temperature for 24 h. The cube specimens were then demolded and cured at 95 ± 5% relative humidity and room temperature for seven days. The aerated slurry mix proportions considered in this experimental program are introduced in Table 1. For the non-aerated slurry (with 0% saponin content), water/cement ratio was between (0.45–0.55).

**Figure 5:**
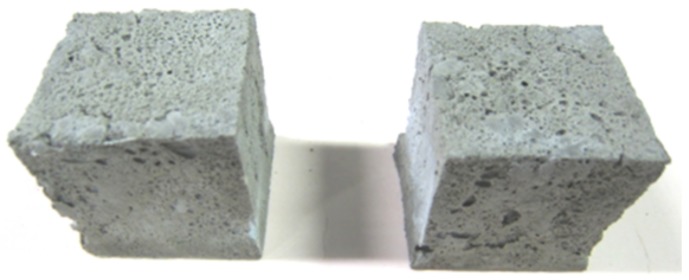
Aerated slurry specimens.

Figure 5 shows examples of the aerated slurry cube specimens that were tested in compression for measurement of the compressive strength of aerated slurry (at seven days of age). The density of aerated slurry was measured by dividing the air-dried weight to the volume of these specimens.

**Figure 6:**
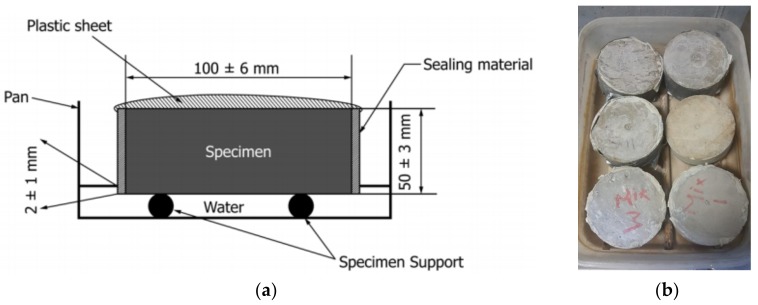
Sorptivity test setup. (**a**) Schematics; (**b**) picture of multiple specimens during the test.

The aerated slurry would be the primary protection of the building interior and also the indigenous insulation to be used within the structural panels against weathering effects. Moisture would be transported through the aerated slurry skins (with chicken mesh reinforcement) via capillary sorption. An experimental study was thus undertaken in order to measure the effects of aeration on the capillary sorptivity of the slurry. Sorptivity tests were performed per ASTM C1585; the specimens used for this purpose were cylinders of 100 mm diameter and 50 mm thickness. A schematic of the test setup is shown in Figure 6a, and a picture of multiple specimens during sorptivity testing is shown in Figure 6b. The specimens were demolded after 24 h of storage in sealed condition and were cured at room temperature until the test age. This test method involves exposure of one flat surface of specimen to water, which the remaining surfaces sealed against moisture loss. Mass gain over time is recorded as a measure of moisture sorption. Sorptivity is expressed in terms of the sorption rate of moisture into the aerated slurry. This test was continued for two days in order to gain further insight into schematic time-history of capillary sorption [31,32].

**Figure 7:**
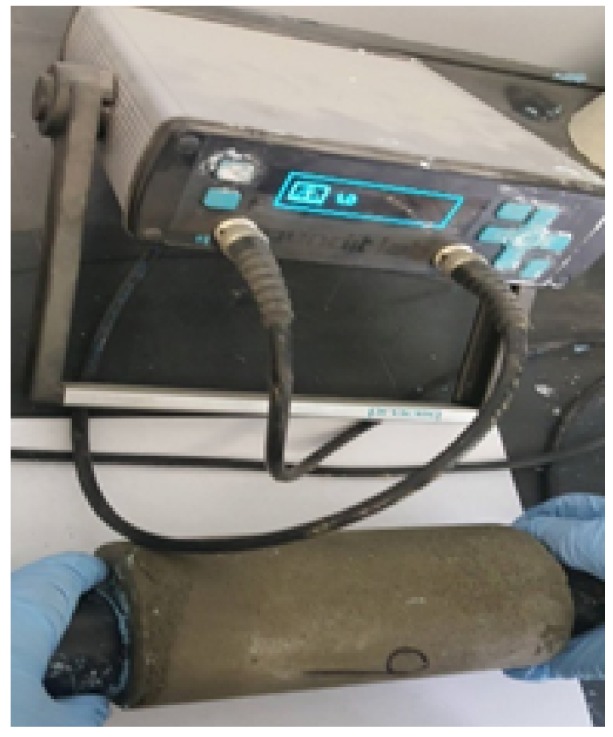
Ultrasound pulse velocity test setup.

The ultrasound pulse velocity (UPV) of aerated slurry was measured nondestructively using a portable equipment (58-E4800 UPV tester, CONTROLS S.p.A, Milan, Itlay). The UPV test setup is shown in Figure 7. In this test, an ultrasonic pulse is generated and transmitted to the surface of concrete through the transmitter transducer. The time taken by the pulse to travel through the aerated slurry, t_us_, is measured by the receiver transducer on the opposite side. The 54 kHz transducers were positioned at the center of each opposing face. The propagation time of the ultrasonic waves transmitted through the 150 mm long cylindrical specimens was measured with accuracy up to 0.1 s. A thin couplant (solid blue kaolin-glycerol paste was used at the interface between transducers and the aerated slurry specimen surfaces to ensure good contact. The pulse travel time (t) from the front side to the rear side was automatically recorded. The ultrasound pulse velocity was measured approximately 50 h after mixing of the aerated slurry [33,34,35]. The specimens used for performance of the ultrasound pulse velocity were prepared from aerated slurry mixes of similar proportions as those used for other experiments; the mixes used for preparation of the ultrasound pulse velocity specimens, however, were not the same as those used for preparation of the specimens used in other tests. All specimens were cured at room temperature and 95 ± 5% relative humidity.

**Figure 8:**
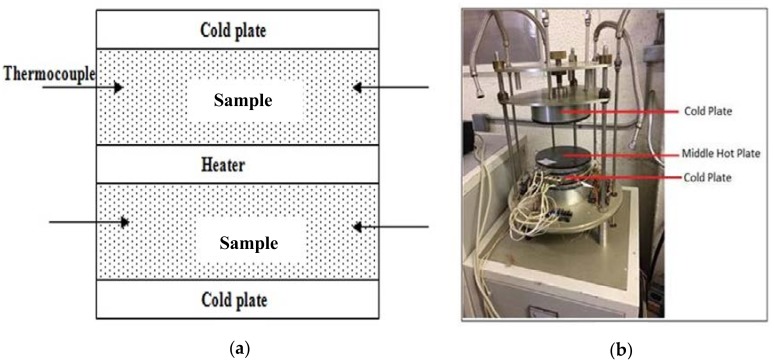
(**a**) Thermal conductivity testing diagram and (**b**) test setup.

The thermal conductivity of aerated slurry test was measured at seven days of age in accordance to ASTM C177 [36]. Aerated slurry cement specimens were oven dried for 24 h at a temperature of 105 ± 5 °C. Figure 8 shows the thermal conductivity testing configuration and test setup. The specimen was placed between hot and cold plates with 40 and 18 °C, respectively, to simulate outdoor and indoor temperatures. Temperatures on the hot and the cold plates as well as the heat flow were recorded versus time over 24 h. The results, after the process reached equilibrium, were used to calculate the thermal conductivity of aerated slurry.

Furthermore, aerated slurry samples were subjected to Scanning Electron Measurement (SEM) observation to evaluate microstructural features. SEM observations were carried out on JCM-5000 NeoScope™ (JEOL Ltd., Tokyo, Japan) at an accelerating voltage of 10–15 kV using a secondary electron (SE) detector. The investigations were conducted on fracture surfaces of the paste of the samples after 28 days. Samples were sputtered with gold before the SEM measurements.

## 3. Experimental Results and Discussion

### 3.1. Compressive Strength 

Table 2 presents the measured values of seven-day compressive strength and density for the aerated slurry mix designs introduced earlier in Table 1. Lower values of density tend to correspond with lower values of compressive strength. This is both because of the rise in air content and also the increase in water/cement ratio for achieving viable fresh mix rheology. Mix 13 with density of 0.9 g/cm^3^ and 5.4 MPa compressive strength at seven days provides a viable balance of density and strength for the targeted ferro-cement application. This paper strongly emphasizes the density of aerated slurry in order to enhance the efficiency of seismic design [37,38] and also to enable manual installation of the building structure.

### 3.2. Sorptivity

**Figure 9:**
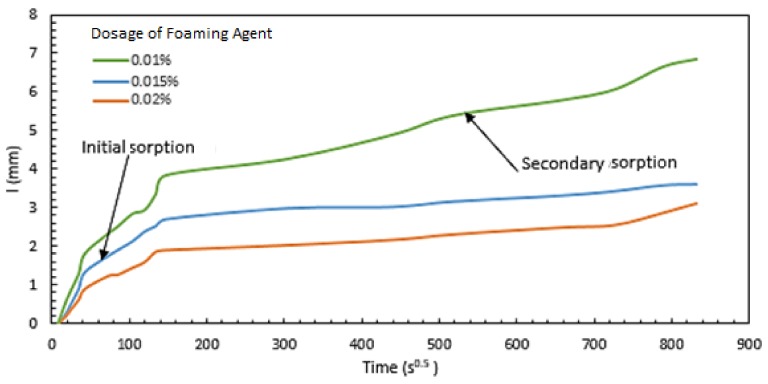
Capillary sorption of aerated slurries versus the square root of time.

The sorptivity test results are presented in Figure 9 as the capillary rise of moisture versus time for aerated slurries prepared with different dosages of the foaming agent (saponin), with water/cement ratio of 0.55. The two higher dosages of foaming agent (0.015% and 0.02%) are observed to produce lower sorption rates and capacities. This is a positive trend, indicating that lowering the density of the slurry via aeration would actually improve its barrier qualities for protecting the building interior as well as the natural insulation against weathering effects. 

The initial sorption rate (*S_i_*) is the slope of the sorption curve shown in Figure 9 up to 6 h; the secondary sorption rate is the slope of the curve after one day. Both these calculations are made using a linear regression analysis (ASTM C1585) [39]:

$$I=S_{i}\times \sqrt{t}+b$$

The resulting values of initial and secondary sorption rate are presented in Table 3 together with the corresponding values of the aerated slurry density. These results confirm that lowering the density of aerated slurry from 1.7 to 1.17–1.3 g/cm^3^ leads to a significant drop in the initial and secondary sorption rates of the slurry. This can be explained by the fact that aeration introduces isolated air bubbles into the slurry, which disrupt the continuity of capillary pores through which sorption occurs [40,41,42,43,44,45]. An overall sorptivity value is also presented in Table 3, which is the slope of a regression line fit into the whole data points (using the above equation). The overall sorptivity values further confirms the drop in sorption rate with reduction of the density of aerated slurry.

**Figure 10:**
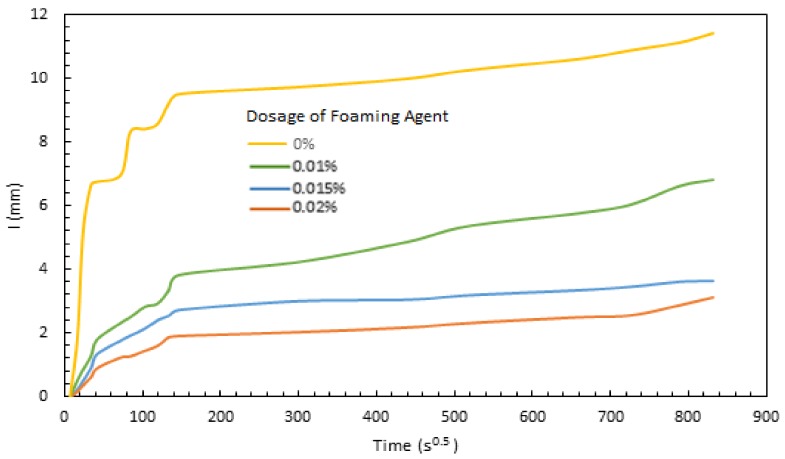
Sorptivity of non-aerated versus aerated slurries.

**Figure 11:**
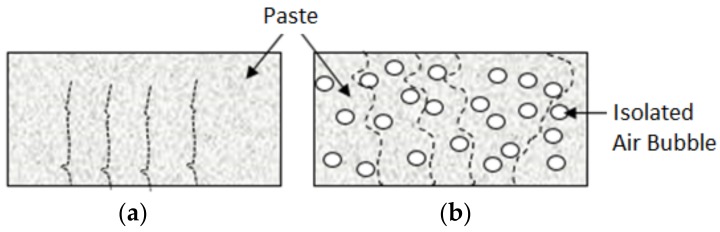
Sorption paths into non-aerated and aerated slurries. (**a**) Non-aerated; (**b**) aerated.

In order to confirm the finding that aeration actually reduces the sorption rate and extent of slurry (i.e., enhances its barrier qualities), tests were also performed on a slurry without any aeration. The sorption test data presented in Figure 10 confirm that aeration reduces the rate and extent of moisture sorption into the slurry. As schematically depicted in Figure 11, introduction of isolated air bubble forces tortuous sorption paths through capillary pores, which reduce the sorption rate of the slurry. The experience with entrained air bubbles indicates that individual air bubbles remain largely empty of water even under long-term exposure to moist conditions. This phenomenon, as well as the reduction in the rate of moisture sorption, explain the drop in the extent of moisture sorption with introduction of individual air bubbles via aeration of slurry.

**Figure 12:**
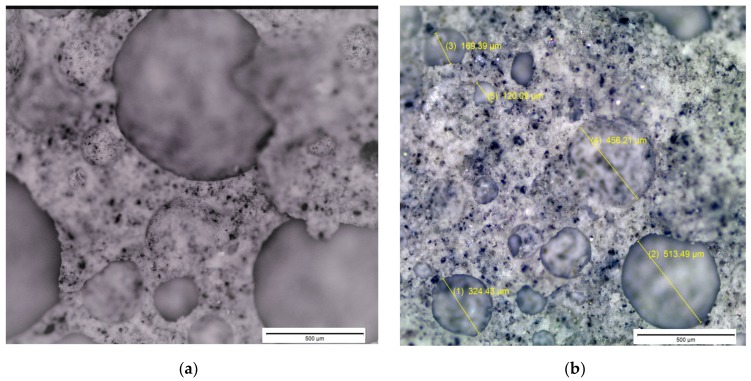
Optical microscope images of sections of aerated slurries with different dosages of the foaming agent (saponin). (**a**) 0.005% foaming agent; (**b**) 0.02% foaming agent.

**Figure 13:**
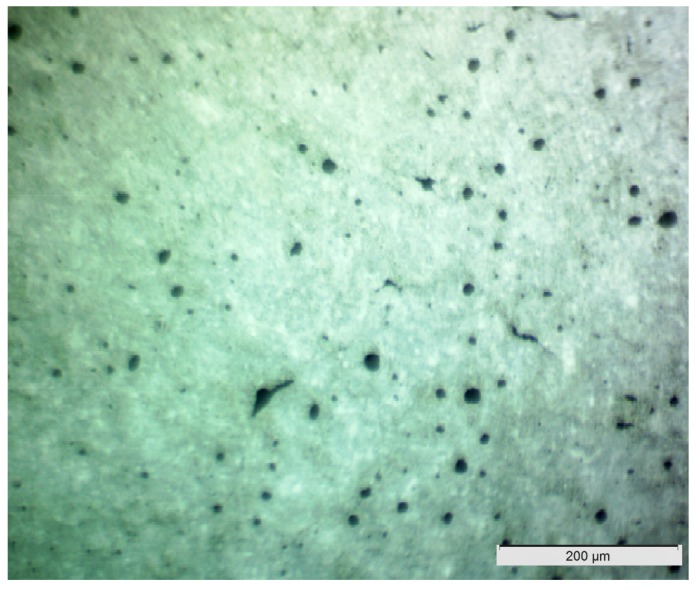
A typical microscopic image of the exterior surface of an aerated slurry with higher compressive strength.

Optic microscope images were obtained from sections of aerated slurries prepared with different dosages of the foaming agent in order to understand the morphology of air bubbles and explain their effects on compressive strength. Figure 12a,b show microscopic images of slurries prepared with 0.005% and 0.02% concentrations of the foaming agent, respectively. A rise in the foaming agent dosage is observed to increase the size (as well as the volume fraction) of air bubble. It should be noted that smaller and regularly formed air bubbles produce higher compressive strengths than coarser and irregularly formed air bubbles [46]. Mechanical properties are strongly influenced by the distribution of pores within the hardened aerated slurry [47,48]. The spherical and distributed nature of foams in Figure 12b with higher air content led to a viable level of compressive strength which was not lower than that provided by the aerated slurry shown in Figure 12b with lower content of irregularly-shaped air bubbles. This observation confirms that microstructural properties are primary factors influencing the material properties of aerated slurry [38]. An example optic microscope image of an exterior surface of an aerated slurry of higher compressive strength is shown in Figure 13, where finer and more uniformly dispersed air bubbles can be observed.

### 3.3. SEM Observations

**Figure 14:**
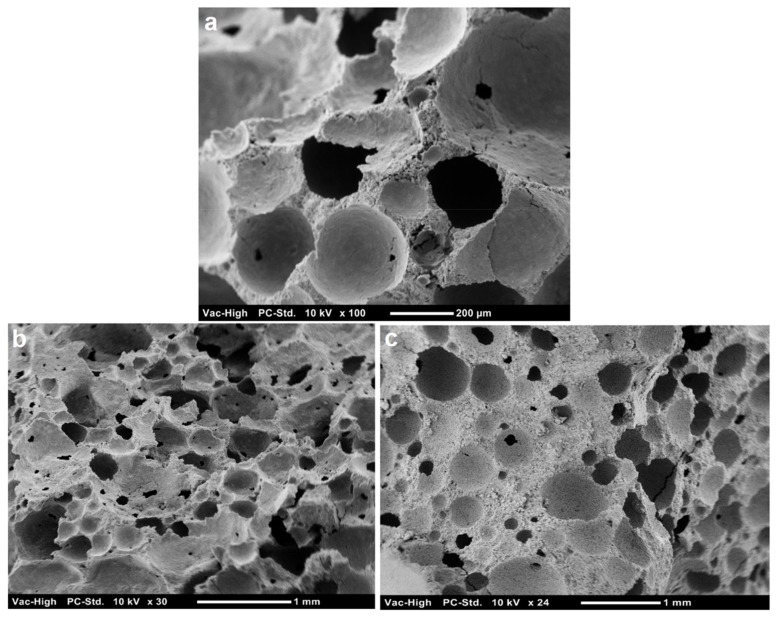
SEM images of the fractured surfaces of foam cements made from slurries after hydration for 28 days with a density of (**a**) 1.0 g/cm^3^ (**b**) 0.75 g/cm^3^ (**c**) 1.3 g/cm^3^.

The spherical geometry of air voids is an important factor influencing the structural and functional properties of aerated binders [49,50]. In addition, the voids should be distributed evenly in the mass to produce homogenous binders of improved performance. Larger voids (macro-pores) would lower the density of aerated slurry but could compromise its mechanical performance. Depending on the type and dosage of the foaming agent, aerated cement slurries may incorporate both micro- and macro-pores [51]. Macro-pores could be formed as a result of the merger of micro-pores. This is because expansion of the matrix upon micro-pore formation generates pressure at the interfaces between micro-pores [52]. Figure 14a shows an SEM image of an aerated slurry after hydration.

Aside from the gel pores (<10 nm) and capillary pores (10 nm to 10 µm), hollow shell pores have been proposed as a third category of intrinsic pores in bulk of hydration products [52]. Hollow shells have range in size from 1 to around 20 µm, about the size of smaller cement grains, embedded in cement gel and channeled to the outside through capillary and gel pores.

An ideal microstructure of aerated cement minimizes the extent of water transport by uniformly distributing the discrete micro-size pores generated by the foaming agent within the cement slurry. The coalescence of many irregular-shaped pores, however, may create a disturbed microstructure, triggering a high degree of water mobility. In order to verify this, two fractured samples of aerated slurries with 0.75 and 1.3 g/cm^3^ bulk densities were examined using a scanning electron microscope (Figure 14b,c, respectively). The image on the right, for the slurry with 1.3 g/cm^3^ bulk density, shows a microstructure of uniformly distributed disjunctive pores. In contrast, a different microstructure was observed for the aerated slurry with 0.75 g/cm^3^ bulk density (left) where an anomalous coalescence developed, yielding a channeled pore structure. This structure allows water to pervade easily through the aerated slurry. A possible explanation for the formation of such an anomalous pore structure is the inclusion of tremendous numbers of air bubble cells using an excess amount of foaming agent, which promotes the coalescence of air bubbles as a result of the collapse of the slurry walls separating these bubbles. 

### 3.4. Ultrasound Pulse Velocity

**Figure 15:**
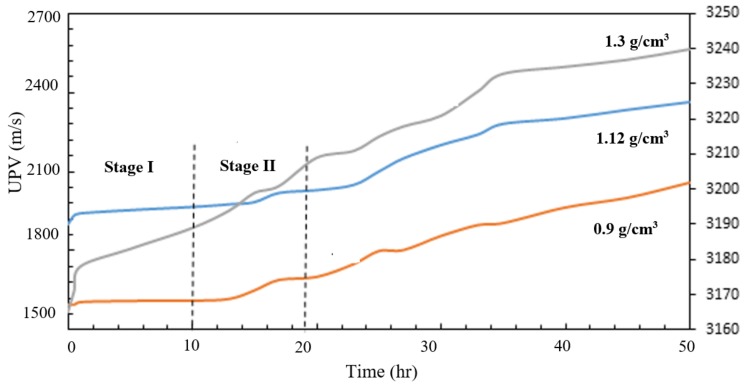
UPV time-history for aerated slurries of different densities.

Ultrasound pulse velocity (UPV) is a simple, nondestructive means of evaluating concrete, which could be used to assess the aerated slurry quality and its development over time. Figure 15 shows the evolution of ultrasound pulse velocity over time (up to 50 h) after mixing for three aerates slurries with different densities. UPV is observed to be higher for aerated slurries of higher density. At earlier ages, lower-density aerated slurries exhibit a minor rise in UPV for more than 10 h while the higher-density slurry exhibits a clear trend towards UPV increase as soon as 1 h after mixing.

**Figure 16:**
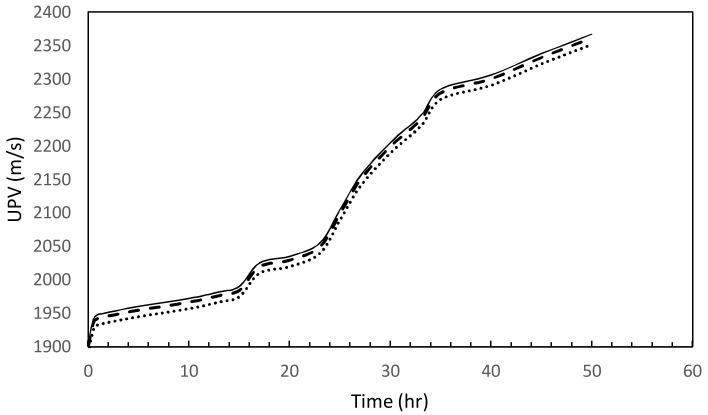
Reproducibility check on three UPV tests for the same mix design (Mix 13).

In order to assess the variability of UPV measurements, three replicated aerated slurry specimens were prepared with the same mix design (Mix 13) and density (0.9 g/cm^3^). The evolution of UPV with time is presented in Figure 16 for the three replicated specimens. The variations in UPV for these three test specimens are less than 6%, pointing at the potential value of UPV as a reliable method of monitoring the quality of aerated slurry (and its evolution with time of curing). 

### 3.5. Thermal Conductivity

**Figure 17:**
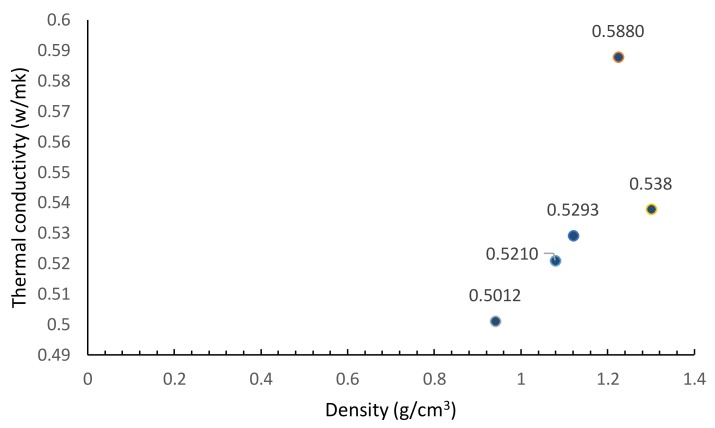
Thermal conductivity of aerated slurries versus their density.

The lightweight aerated slurry is expected to make some contributions towards thermal insulation of the building. The measured values of thermal conductivity are shown in Figure 17 versus the density of aerated slurries. As expected, slurries of lower density offer lower values of thermal conductivity [8,53,54]. Air bubbles act as barriers against thermal conduction; the isolated air bubbles do not make any significant contributions to heat transfer via convection [7].

## 4. Conclusions

Aerated slurry is developed as a lightweight matrix for production of cementitious composites embodying reinforcement of high specific surface area for structural applications. A highly flowable slurry is needed for thorough infiltration of the structural volume that is congested with fine reinforcement systems. This work developed and characterized an aerated slurry comprising a cementitious material of relatively high water/cement ratio that incorporated a foaming agent (saponin). High-speed mixing of the mixing water incorporating saponin produces the foamed water that is then used to prepare the aerated slurry by mixing with cement. The following conclusions were derived by conducting an experimental program on slurries of various densities (adjusted by varying the saponin content).

1. While lowering the density of the aerated slurry by increasing the dosage of foaming agent tends to lower its compressive strength, this relationship is not consistent. Production of fine, spherical and uniformly distributed air bubbles in aerated slurry favors achievement of higher compressive strengths.
2. Aeration of slurry benefits its moisture barrier qualities, which benefits its durability. The isolated air bubbles in aerated slurry seem to act as barriers against capillary sorption of moisture into the slurry, thus forcing tortuous diffusion paths. The extent of moisture sorption by slurry also decreases with increasing air content. This could be attributed to the tendency of the isolated air bubbles to remain largely filled with air when the aerated slurry is exposed to moisture.
3. Aeration of the cement slurry significantly reduces its thermal conductivity, which benefits the energy-efficiency of building systems. The low thermal conductivity of air in the bubbles introduced via aeration, and the lack of effective convection due to the isolated nature of air bubbles, explain the benefits of aeration towards the insulation value of aerated slurry.
4. Ultrasound pulse velocity provides an effective nondestructive means of controlling the quality of aerated slurry and its evolution over time. This method can be conveniently implemented in field conditions for assessing the quality of aerated slurry and its evolution over time.

## Figures and Tables

**Table 1:** The aerated slurry mixes proportions considered in this investigation.

Mix	Saponin Dosage (by Weight of Cement)	Water/Cement Ratio
1	0.005%	0.45
2	0.01%	
3	0.02%	
4	0.005%	0.50
5	0.01%	
6	0.02%	
7	0.005%	0.55
8	0.01%	
9	0.015%	
10	0.02%	
11	0.025%	
12	0.03%	
13	0.02%	0.6
14	0.025%	

**Table 2:** Mix designs and performance characteristics of the aerated slurry.

Mix	Seven-Day Compressive Strength, MPa	Density, g/cm^3^
1	10.7	1.9
2	8.2	1.5
3	6.3	1.4
4	14.1	1.2
5	10.5	1.81
6	9.2	1.3
7	13.3	1.6
8	11.1	1.7
9	9.4	1.3
10	6.4	1.17
11	2.4	0.65
12	1.2	0.8
13	5.4	0.9
14	7.1	1.12

**Table 3:** Sorption rates and densities of slurries prepared with different dosages of the foaming agent.

Dosage of Foaming Agent %	0.01%	0.015%	0.02%
Initial sorption rate, mm/$\sqrt{s}$	0.0242	0.0188	0.0132
Secondary sorption rate, mm/$\sqrt{s}$	0.0044	0.0013	0.0019
R^2^ (Regression value)	0.951	0.950	0.958
Density, g/cm^3^	1.7	1.3	1.17
Sorptivity, mm/min^0.5^	0.75	0.5	0.34
